# Research on stress field inversion and large deformation level determination of super deep buried soft rock tunnel

**DOI:** 10.1038/s41598-024-62597-9

**Published:** 2024-06-03

**Authors:** Baojin Zhang, Zhongsheng Tan, Jinpeng Zhao, Fengxi Wang, Ke Lin

**Affiliations:** 1 Key Laboratory of Urban Underground Engineering of Ministry of Education, Beijing Jiaotong University, Beijing, 100044 China; 2 China Railway 16th Bureau Group 3rd Corporation Limited, Huzhou, 313000 China; 3 State Key Laboratory of Hydroscience and Engineering, Tsinghua University, Beijing, 100084 China

**Keywords:** High geo-stress, Large deformation tunnel, GS-XGBoost algorithm, Geo-stress inversion, Large deformation level, Engineering, Civil engineering

## Abstract

Understanding the characteristics and distribution patterns of the initial geo-stress field in tunnels is of great significance for studying the problem of large deformation of tunnels under high geo-stress conditions. This article proposes a ground stress field inversion method and large deformation level determination based on the GS-XGBoost algorithm and the Haba Snow Mountain Tunnel of the Lixiang Railway. Firstly, the hydraulic fracturing method is used to conduct on-site testing of tunnel ground stress and obtain tunnel ground stress data. Then, a three-dimensional model of the Haba Snow Mountain Tunnel will be established, and it will be combined with the GS-XGBoost regression algorithm model to obtain the optimal boundary conditions of the model. Finally, the optimal boundary condition parameters are substituted into the three-dimensional finite-difference calculation model for stress calculation, and the distribution of the in-situ stress field of the entire calculation model is obtained. Finally, the level of large deformation of the Haba Snow Mountain Tunnel will be determined. The results show that the ground stress of the tunnel increases with the increase of burial depth, with the maximum horizontal principal stress of 38.03 MPa and the minimum horizontal principal stress of 26.07 MPa. The Haba Snow Mountain Tunnel has large deformation problems of levels I, II, III, and IV. Level III and IV large deformations are generally accompanied by higher ground stress (above 28 MPa) and smaller surrounding rock strength. The distribution of surrounding rock strength along the tunnel axis shows a clear "W" shape, opposite to the surface elevation "M" shape. It is inferred that the mountain may be affected by geological structures on both sides of the north and south, causing more severe compression of the tunnel surrounding rock at the peak.

## Introduction

With the continuous development of the southwestern region of China, the number of tunnel construction is increasing, and the constructed tunnels are developing towards a trend of "long, large, and deep"^1,2^. In addition, the terrain in the southwestern region of China is undulating, and the tunnel construction environment is complex, especially when the tunnel burial depth is large, resulting in high geo-stress^3,4^. Encountering weak rock masses will bring serious large deformation disasters, leading to adverse consequences such as support failure, delayed construction period, and increased cost^5^. The state and distribution of the initial geo-stress field in tunnels are an important basis for studying tunnel support design under high geo-stress conditions, which helps designers take reasonable preventive measures and reduce construction risks^6^. Due to the influence of various factors such as terrain, lithology, and geological structure on the initial stress field, terrain affects the distribution of tunnel loads. Rock lithology determines mechanical indicators such as elastic modulus and strength of rocks, and geological structures such as faults and folds can lead to local stress concentration in tunnels. The coupling of terrain, lithology, geological structure, and other factors leads to the complex distribution of the underground initial stress field. It is difficult to obtain the initial stress field characteristics of the tunnel^7^.

In the survey stage of tunnel engineering, in-situ stress testing is an important content of the survey. With the development of tunnel construction, researchers have proposed various in-situ stress testing methods, such as the hydraulic fracturing method^8,9^ and the stress relief method^10,11^. These two methods are widely used in practice. Liu et al. measured the in-situ stress of the Yangshan tunnel using the hydraulic fracturing method^12^. The test results indicate that the main direction of the maximum horizontal principal stress near the tunnel is N57°–N74° E, with an average value of N65.5° E. The test results provide data support for precise design and scientific decision-making in tunnel engineering. Tan et al. used the hollow inclusion stress relief method to test the in-situ stress of the tunnels of the China-Laos railway under the special geological influence of the Luang Prabang suture zone^13^. The test results show that the inclination angle of the maximum principal stress (*σ*_1_) in the tunnel and the inclination angle of the intermediate principal stress (*σ*_2_) are close to horizontal, while the minimum principal stress (*σ*_3_) is close to vertical. The maximum and medium principal stress intersect with the tunnel axis in the horizontal direction, and the lateral pressure coefficient exceeds 1.5. The actual deformation of the tunnel exhibits significant horizontal convergence deformation, which also verifies the accuracy of the test results. However, due to their high cost and complex operation, these measurement methods usually only select individual measurement points within a limited range for testing, resulting in highly discrete results^14^. The test results cannot represent the in-situ stress characteristics within the entire tunnel area. Therefore, the guiding significance of these methods is limited to ultra-deep and long-distance tunnels.

Therefore, researchers combine numerical analysis methods with measured data, and based on a small amount of measured data, using scientifically effective numerical inversion analysis methods can obtain a large range of in-situ stress field data and stress distribution characteristics, which is an economically effective method^15^. Typical initial geo-stress inversion analysis methods include displacement inversion analysis^16,17^, multiple regression analysis^18^, and intelligent algorithms. The displacement inversion analysis method adjusts the rock mass parameters and in-situ stress field multiple times to approximate the actual tunnel displacement in the model, which has good guiding significance during tunnel construction^19^. Multiple regression analysis methods mainly include linear regression, nonlinear regression, ridge regression, and support vector regression^20^. After determining the regression coefficient, the initial geo-stress field in the tunnel site area can be easily and quickly obtained, which has been widely used^21^. Intelligent algorithms, including neural network algorithms^22,23^ and genetic algorithms^24^, have significant advantages in nonlinear analysis and fuzzy recognition^25^. Many scholars have studied the characteristics of in-situ stress near tunnel faults through in-situ stress inversion. Xu et al. inverted the tectonic stress field near the fault in Shihao coal mine through multiple linear regression^26^. The results indicate a significant variation in the magnitude of ground stress near the fault, and the impact area of the fault on ground stress is mainly within 100 m. Outside the influence range of the fault, the maximum principal stress and maximum shear stress of the rock layer decrease with the increase of rock strength, and the maximum values of the maximum principal stress and maximum shear stress of each rock layer near the fault are obtained. This study accurately inverted the tectonic stress field near the fault, which has a certain guiding significance for the safe mining of coal mines. Li et al. collected a large amount of in-situ stress data near the Yishu fault zone and analyzed the characteristics of the in-situ stress field near the fault zone through linear regression^27^. Research has found a significant difference in the in-situ stress distribution on both sides of the fault zone. The maximum horizontal principal stress direction on the west side of the fault zone is mainly NEE-SWW, while the maximum principal stress direction on the east side is mainly NWW-SEE.

**Figure 1:**
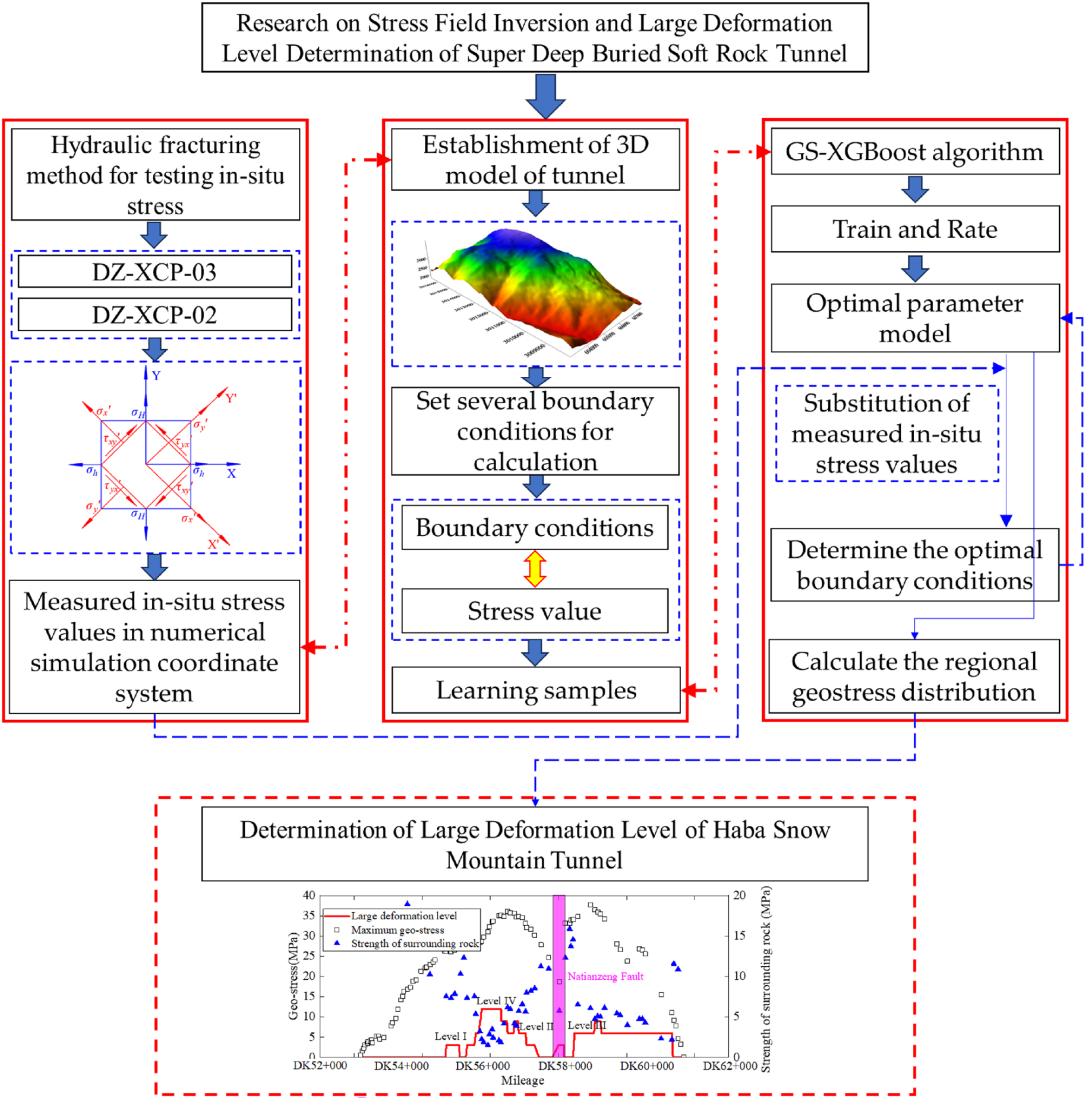
The technical roadmap.

This study relies on the Haba Snow Mountain Tunnel of the Lixiang Railway and first tests the ground stress of the tunnel using the hydraulic fracturing method. Secondly, through numerical simulation methods combined with the GS-XGBoost algorithm, the in-situ stress field of the tunnel site is inverted. Combined with the on-site in-situ stress test results, the effectiveness of the numerical model's in-situ stress inversion information is verified, and the numerical values and characteristics of the tunnel site's in-situ stress are obtained. Then, the distribution pattern of ground stress in the longitudinal direction of the tunnel will be analyzed. Finally, the level of large deformation of the Haba Snow Mountain Tunnel will be determined. The technical roadmap for this study is shown in Fig. 1.

## Ground stress field inversion theory

### Factors affecting the geo-stress field

**Figure 2:**
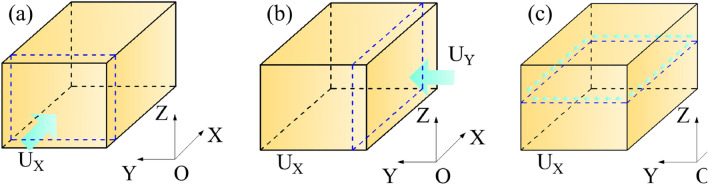
Regional model of geo-stress inversion research: (**a**) X-direction compressive tectonic movement; (**b**) Y-direction compressive tectonic movement; (**c**) XY plane shear tectonic movement.

For general geological bodies, the geo-stress field mainly considers the influence of the self weight of surrounding rock and tectonic movement^28^. The self weight of the surrounding rock is mainly influenced by the density of the rock mass and the local gravitational acceleration, and tectonic movement is mainly reflected in compression and shear^29^. Previous studies have shown no necessary connection between horizontal principal stress and vertical stress in rock masses. The lateral pressure coefficient is not the same in different directions, and the horizontal principal stress is mainly related to geological tectonic movement^30^. From practical engineering experience, it can be inferred that the gravity of the rock mass itself and the geological tectonic process are the main factors affecting the initial geo-stress^31^. Therefore, this geo-stress inversion mainly considers the basic influencing factors of the initial geo-stress field, including ① Self-weight stress: The results of in-situ stress testing and a large number of practical engineering geo-stress regression studies have shown that self weight stress is one of the main causes of the formation of rock mass geo-stress field. ② Horizontal uniform compression tectonic movement in the east–west X-direction (see Fig. 2a). ③ Horizontal uniform compression tectonic movement in the north–south Y-direction (see Fig. 2b). ④ Shear deformation tectonic movement (see Fig. 2c).

### Hydraulic fracturing method

**Figure 3:**
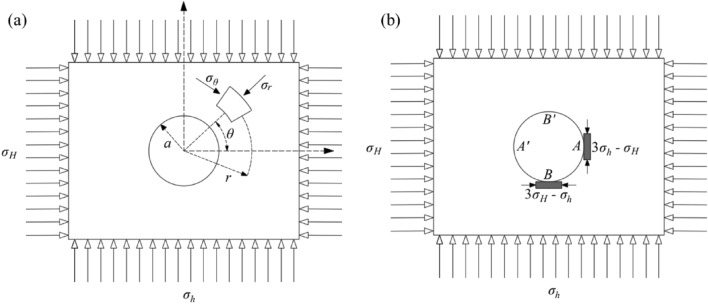
Mechanical model for in-situ stress testing using hydraulic fracturing method: (**a**) a stress model for circular hole micro element plane; (**b**) stress concentration on the wall of a circular hole.

The hydraulic fracturing method testing is based on elastic mechanics with three assumptions as prerequisites: ① The rock is linearly elastic and isotropic. ② The rock is intact, and the fracturing fluid is impervious to the rock. ③ The vertical stress direction of the rock layer is parallel to the drilling axis. Obtain the in-situ stress value of the measuring point by injecting water and pressurizing^32^. Figure 3 shows the stress distribution in the rock mass containing boreholes *σ*_H_ is the maximum horizontal principal stress, *σ*_h_ is the minimum horizontal principal stress^33^.

By substituting the stress state of element M in Fig. 3 into the wall of the circular hole, the stress state at any point on the wall of the circular hole can be obtained, as shown in Eq. (1).

1 $$\left\{ \begin{gathered} \sigma_{r} = 0 \hfill \\ \sigma_{\theta } = \left( {\sigma_{H} + \sigma_{h} } \right) - \left( {2\sigma_{H} - \sigma_{h} } \right)\cos 2\theta \hfill \\ \tau_{r\theta } = 0 \hfill \\ \end{gathered} \right.$$

In Eq. (1), *σ*_r_ and *σ*_θ_, The radial and tangential stress on the hole wall are represented, respectively. According to Eq. (1), the stress Eq. (2) for four points *A*、*A'*、*B* and *B'* on the circular hole wall can be obtained. Based on this Equation, the in-situ stress value of the measurement point can be obtained by substituting multiple injection pressures.

2 $$\left\{ \begin{gathered} \sigma_{A} = \sigma_{{A^{\prime}}} = 3\sigma_{h} - \sigma_{H} \hfill \\ \sigma_{B} = \sigma_{{B^{\prime}}} = 3\sigma_{H} - \sigma_{h} \hfill \\ \end{gathered} \right.$$

During the testing process, for the first time, water is injected and pressurized to the critical rupture pressure *P*_b_ in the dividing section. Suppose the critical rupture water pressure at the measuring point is greater than the ultimate tensile strength Thf of the rock. In that case, tensile rupture will occur along the minimum tangential stress position *A* and the symmetric point *A'*. It will propagate along the direction of the vertical minimum principal stress. Considering the pore pressure *P*_0_ within the rock, the stress relationship can be expressed as Eq. (3).

3 $$P_{b} = 3\sigma_{h} - \sigma_{H} + T_{hf} - P_{0}$$

After the hole wall ruptures, continue injecting water and pressurize; the crack will further expand towards deeper layers. Ensure that the fracturing circuit is sealed, that water injection is stopped, and that the cracks will close under geo-stress. At this point, the equilibrium pressure at the critical closure state of the crack is the instantaneous closure pressure *P*_s_, equal to the minimum horizontal principal stress measured in the borehole.

4 $$\sigma_{h} = P_{s}$$

After injecting water and pressurizing the test partition section again, the crack reopened. As the rock had already ruptured at this time, the tensile strength *T*_*hf*_ was 0. Based on the relationship between the critical fracture pressure, re-tension pressure, and instantaneous closure pressure of the crack, the maximum horizontal principal stress calculation Eq. (5) can be obtained by substituting it into Eq. (3), where *P*_r_ is the again open pressure of the crack.

5 $$\sigma_{H} = 3P_{s} - P_{r} - P_{0}$$

According to the aforementioned assumption ③, the vertical stress *σ*_v_ is equal to the self-weight of the soil cover. Thus, the magnitude of the geo-stress in the separation section of the hydraulic fracturing test hole can be obtained.

### GS-XGBoost algorithm

**Figure 4:**
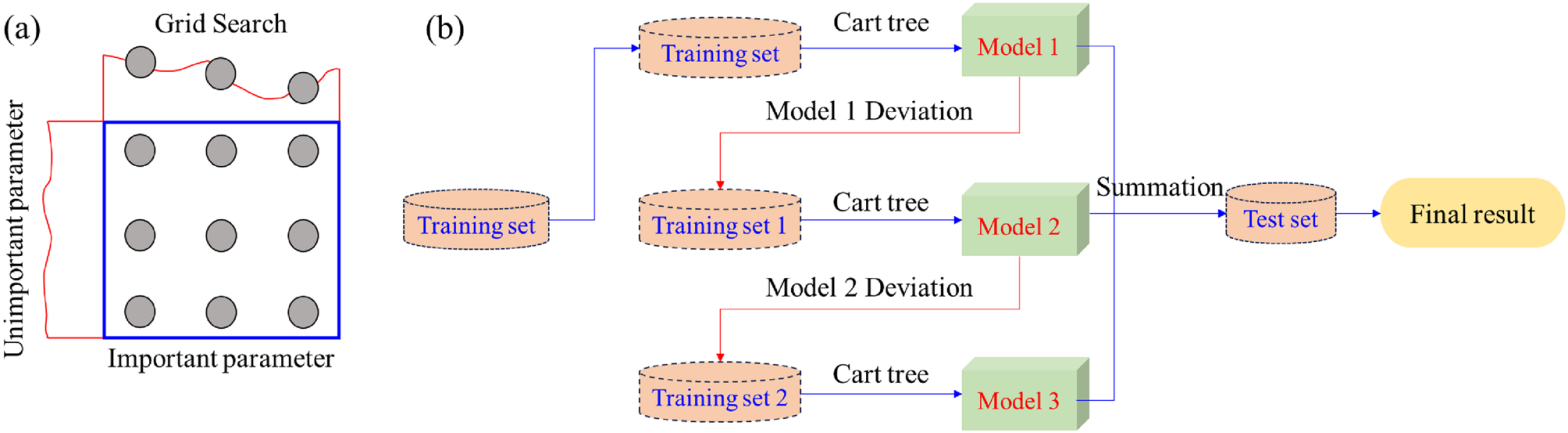
The principle of GS-XGBoost Algorithm: (**a**) the principle of Grid Search; (**b**) the principle of XGBoost Algorithm.

GS (Grid Search) is an exhaustive parameter tuning method^34^. Among all the candidate parameters, adjust the parameters in order of step size and try every possibility through loop traversal to find the parameter with the highest accuracy on the validation set from all the parameters. The best performing parameter is the final result. Grid search can ensure that the most accurate parameter is found within the specified parameter range, as it traverses all possible combinations of parameters^34^. The principle of GS-XGBoost Algorithm is shown in Fig. 4. The principle of Grid Search is shown in Fig. 4a, and the principle of XGBoost Algorithm is shown in Fig. 4b.

The XGBoost algorithm improves the Gradient Ascension Decision Tree Theory (GBDT). The XGBoost algorithm and Gradient Boosting Decision Tree Theory (GBDT) are ensemble learning methods based on Boosting thinking^35^. Gradient Boost Decision Tree Theory (GBDT) only uses first-order derivatives for optimization, while the XGBoost algorithm performs second-order Taylor expansion on the function, using both first-order and second-order derivatives. Therefore, the XGBoost algorithm has advantages such as high accuracy, fast running speed, the ability to process large-scale data, and the ability to customize objective functions. The XGBoost algorithm is an integrated algorithm that uses classification and regression trees as the basic models. In XGBoost, the predicted value of the sample is the sum of the predicted values of each tree in *K* trees. Define the function as:

6 $$\hat{y}^{(k)} = \sum\limits_{k = 1}^{K} {f_{k} } \left( {x_{i} } \right)$$

Among them, *f*_k_ is the k-th decision tree. *x*_i_ is the characteristic value corresponding to sample *i. f*_k_(*x*_i_) is the leaf weight, which denotes the predicted value of the k-th tree for sample *i*. The prediction result of XGBoost is the sum of the leaf weight weights of *K* trees. The objective function of the XGBoost algorithm is:

7 $$L(\emptyset ) = \sum\limits_{i = 1}^{n} l \left( {y_{i} ,\hat{y}_{i} } \right) + \sum\limits_{k = 1}^{K} \Omega \left( {f_{k} } \right)$$

This loss function consists of two components: the loss value $\sum l \left( {y_{i} ,\hat{y}_{i} } \right)$ and the regularization term $\Omega \left( {f_{k} } \right)$, in which the loss value measures the difference between the true label *y*_i_ and the predicted value $\hat{y}_{i}$, and the regularization term controls the complexity of the model. The residuals of the last prediction need to be fitted to the tree generated each time; i.e., when a tree is generated, the predicted values are:

8 $$\hat{y}^{(t)} = \sum\limits_{k = 1}^{t} {f_{k} } \left( {x_{i} } \right) = \hat{y}^{(t + 1)} + f_{t} \left( {x_{i} } \right)$$

Therefore, the objective function can be rewritten as:

9 $$L^{(t)} = \Omega \left( {f_{t} } \right) + \sum\limits_{i = 1}^{n} l \left( {y_{i}^{t} ,\hat{y}^{(t + 1)} + f_{t} \left( {x_{i} } \right)} \right)$$

The next step is to find *f*_t_, which is the minimization of the objective function. The second-order Taylor expansion of the objective function is as follows:

10 $$L^{(t)} = \Omega \left( {f_{t} } \right) + \sum\limits_{i = 1}^{n} {\left[ {l\left( {y_{i}^{t} ,\hat{y}^{(t + 1)} + \frac{1}{2}h_{i} f_{t}^{2} \left( {x_{i} } \right) + g_{i} f_{t} \left( {x_{i} } \right)} \right)} \right]}$$

where *g*_i_ is the first derivative and *h*_i_ is the second derivative:

11 $$g_{i} = \partial_{{\hat{y}^{(t - 1)} }} l\left( {y_{i} ,\hat{y}_{i}^{(t - 1)} } \right),h_{i} = \partial_{{\hat{y}^{(t - 1)} }}^{2} l\left( {y_{i} ,\hat{y}_{i}^{(t - 1)} } \right)$$

The predicted value of the (t − 1) th tree and the residual of y do not affect the optimization of the objective function and can be directly deleted. Therefore, the objective function is simplified as:

12 $$L^{(t)} = \Omega \left( {f_{t} } \right) + \sum\limits_{i = 1}^{n} {\left[ {f_{t} \left( {x_{i} } \right)g_{i} + \frac{1}{2}f_{t}^{2} \left( {x_{i} } \right)h_{i} } \right]}$$

The regularization term $\Omega \left( {f_{t} } \right)$ is:

13 $$\Omega \left( {f_{t} } \right) = \gamma T + \frac{1}{2}\lambda \left\| { \cdot \omega } \right\|^{2} = \gamma T + \frac{1}{2}\lambda \sum\limits_{j = 1}^{T} {\omega_{j}^{2} }$$

where *T* is the number of leaf nodes and $\omega_{j}^{{\phantom{0}}}$ represents the predicted value of the *j-*th leaf node. After substituting the objective function:

14 $$\begin{gathered} L^{\left( t \right)} = \sum\limits_{i = 1}^{n} {\left[ {g_{i} f_{t} \left( {x_{i} } \right) + \frac{1}{2}h_{i} f_{t}^{2} \left( {x_{i} } \right)} \right]} + \Omega \left( {f_{t} } \right) \hfill \\ = \sum\limits_{i = 1}^{n} {\left[ {g_{i} \omega_{{q\left( {x_{i} } \right)}} + \frac{1}{2}h_{i} \omega_{{q\left( {x_{i} } \right)}}^{2} } \right]} + \gamma T + \lambda \frac{1}{2}\sum\limits_{J = 1}^{T} {\omega_{J}^{2} } \hfill \\ = \sum\limits_{j = 1}^{T} {\left[ {\left( {\sum\limits_{{i \in I_{j} }} {g_{i} } } \right)\omega_{j} + \frac{1}{2}\left( {\sum\limits_{{i \in I_{j} }} {h_{j} } + \lambda } \right)\omega_{j}^{2} } \right]} + \gamma T \hfill \\ \end{gathered}$$

where *T* is the number of leaf nodes in the decision tree *f*_t_, and *I*_*j*_ represents the combination of all sample indexes belonging to leaf node *j.* Let $G_{j} = \sum {_{i \in I_{j}} } g_{i}$, $H_{j} = \sum {_{i \in I_{j} } } h_{i}$, the objective function is then

15 $$L^{(t)} = \left[ {\sum\limits_{i = 1}^{n} {G_{j} } \omega_{j} + \frac{1}{2}\left( {H_{j} + \lambda } \right)\omega_{j}^{2} } \right] + \gamma T$$

Assuming that the structure of the decision tree is known, and by setting the derivative of the objective function relative to $\omega_{j}^{{\phantom{0}}}$ be 0, the prediction on each leaf node can be obtained under the condition of minimizing the loss function as

16 $$\omega_{j}^{*} = - \frac{{G_{j} }}{{H_{j} + \lambda }}$$

The minimum value of the loss function can be found by bringing the predicted values into it:

17 $$L_{j}^{*} = - \frac{1}{2}\sum\limits_{j = 1}^{T} {\frac{{G_{j}^{2} }}{{H_{j} + \lambda }}} + \gamma T$$

It is easy to calculate the difference of the loss function before and after splitting:

18 $${\text{ Gain }} = \frac{{G_{L}^{2} }}{{H_{L} + \lambda }} + \frac{{G_{R}^{2} }}{{H_{R} + \lambda }} - \frac{{\left( {G_{L} + G_{R} } \right)^{2} }}{{H_{L} + H_{R} + \lambda }} - \gamma$$

XGBoost constructs the decision tree based on the difference obtained from Eq. (18), and by traversing the value cases of all features, a node is selected for splitting when the difference between the value before and after the loss function reaches the maximum value. In addition, the difference in the loss function before and after splitting must be positive, which can be considered to play the role of prehearing.

### Inversion process of in-situ stress field

**Figure 5:**
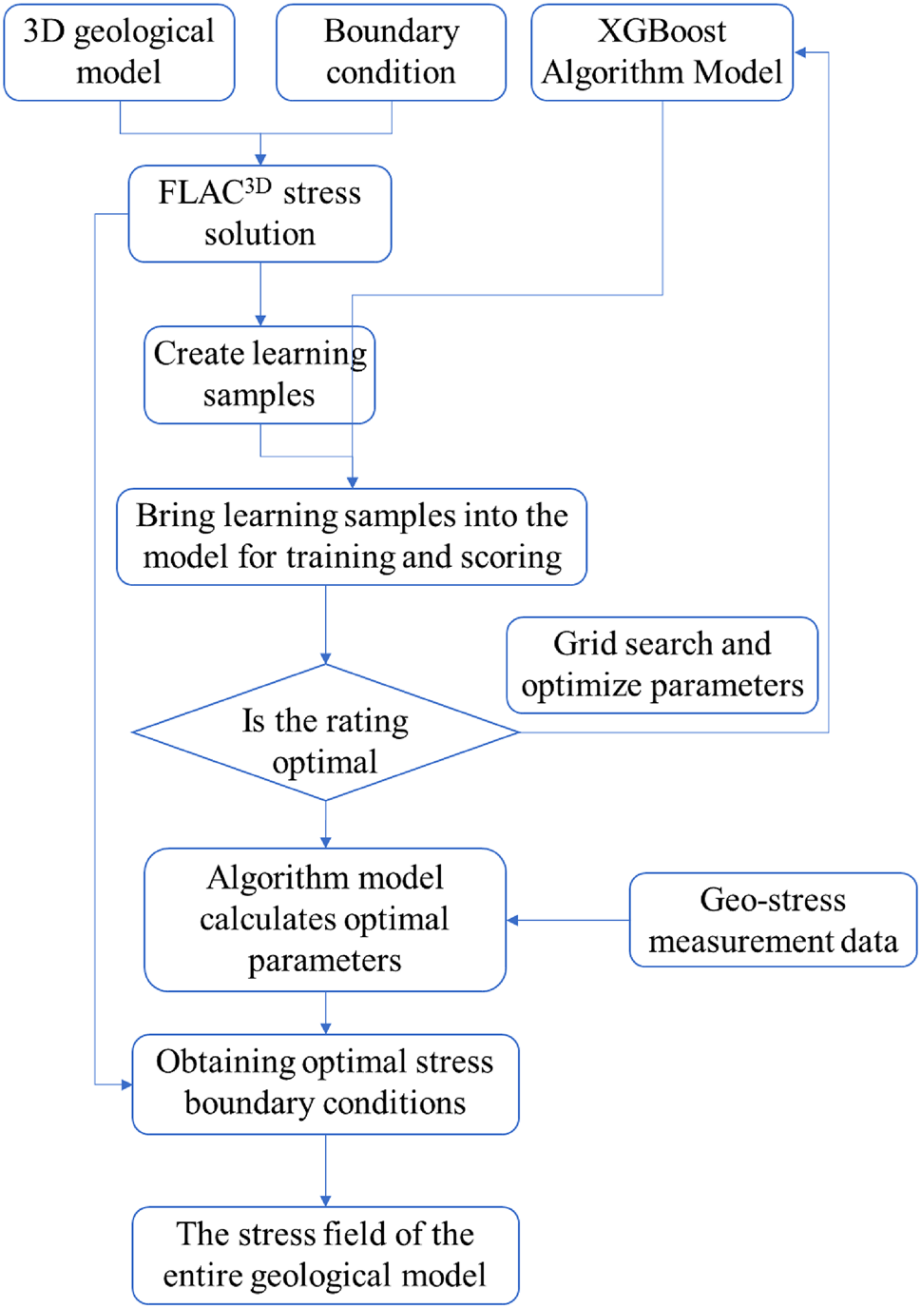
In-situ stress inversion method based on GS-XGBoost regression algorithm.

The specific process of in-situ stress field inversion is shown in Fig. 5. ① Conduct data research and on-site investigation to obtain basic information such as topographic maps, borehole histograms, structural distribution, and physical properties of rock layers. Use mapping software to establish a three-dimensional geological model. ② Import the geological model into the finite element calculation software and establish a three-dimensional finite element calculation model through steps such as material parameter definition and mesh division. Then, based on the comprehensive influencing factors of the geo-stress field, several sets of boundary condition combinations are set up and applied to the finite element model for stress calculation. ③ Obtain the simulated stress magnitude of the measured point positions, establish corresponding relationships between the stress states and boundary conditions at all positions, and create learning samples. ④ Write a GS-XGBoost regression algorithm model using Python, train and score the learning samples into the model, adjust the model parameters based on the score, and find the optimal parameter model. ⑤ Substitute the measured in-situ stress values into the optimal parameter model and obtain the predicted values as the corresponding optimal boundary conditions. ⑥ Substitute the optimal boundary condition parameters into the three-dimensional finite element calculation model for stress solution and obtain the distribution of the in-situ stress field of the entire calculation model.

## Inversion of ground stress field in Haba Snow Mountain Tunnel

### Establishment of a three-dimensional model for the Haba Snow Mountain Tunnel

**Figure 6:**
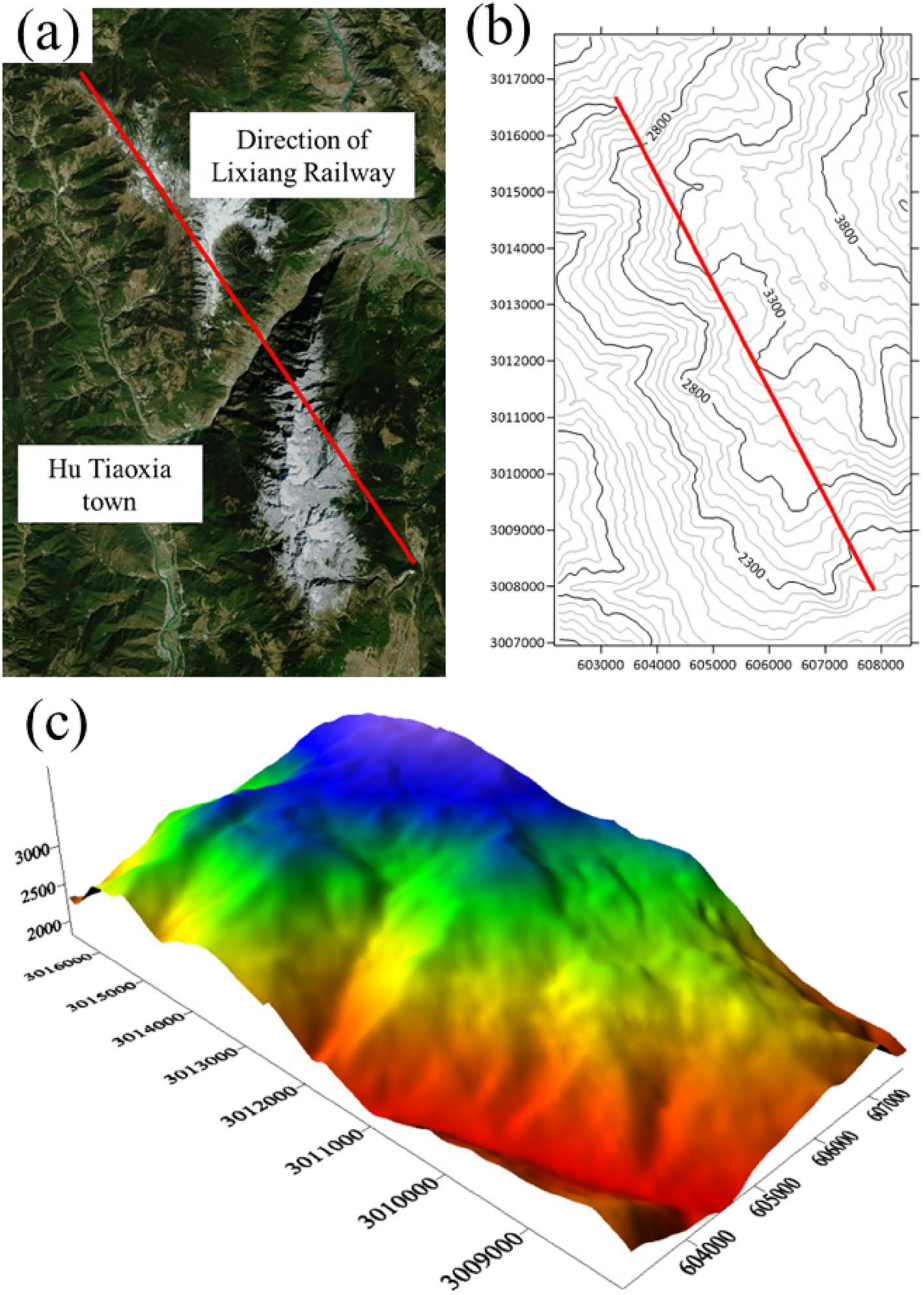
Study area of geo-stress inversion: (**a**) Satellite map of the study area; (**b**) Contour map of the study area; (**c**) Three-dimensional elevation map in study area.

By using numerical simulation methods to invert the geo-stress field in the tunnel site area and combining with the results of on-site geo-stress drilling tests, the effectiveness of the numerical model's geo-stress inversion information is verified, and the distribution pattern of longitudinal geo-stress in the tunnel is obtained. The research area mainly focuses on the rectangular range of the entire Haba Snow Mountain Tunnel, as shown in Fig. 6a, with longitude of 100.039605680°–100.090074125° and latitude of 27.177790207°–27.256411118°. Tunnel entrance coordinates (E607800, N3008000), exit coordinates (E603244, N3016375). Obtain satellite images of the tunnel surface through the 91 satellite map assistant, use the "elevation download" function to frame the research area, use the 15th level elevation, sampling distance of 67.97 m, select the "Xi'an 80 coordinate system Gaussian projection" for coordinate projection, and save it as a Surfer grid file. Using the contour drawing function in Surfer software, plot the elevation information in the 91 satellite map assistant, as shown in Fig. 6b. The horizontal and vertical coordinates in the figure are the local coordinate system automatically generated by Surfer software, and the red thick lines represent the tunnel position. Further, the elevation information is processed, and the three-dimensional transformation is performed, with the surface elevation shown in Fig. 6c.

**Figure 7:**
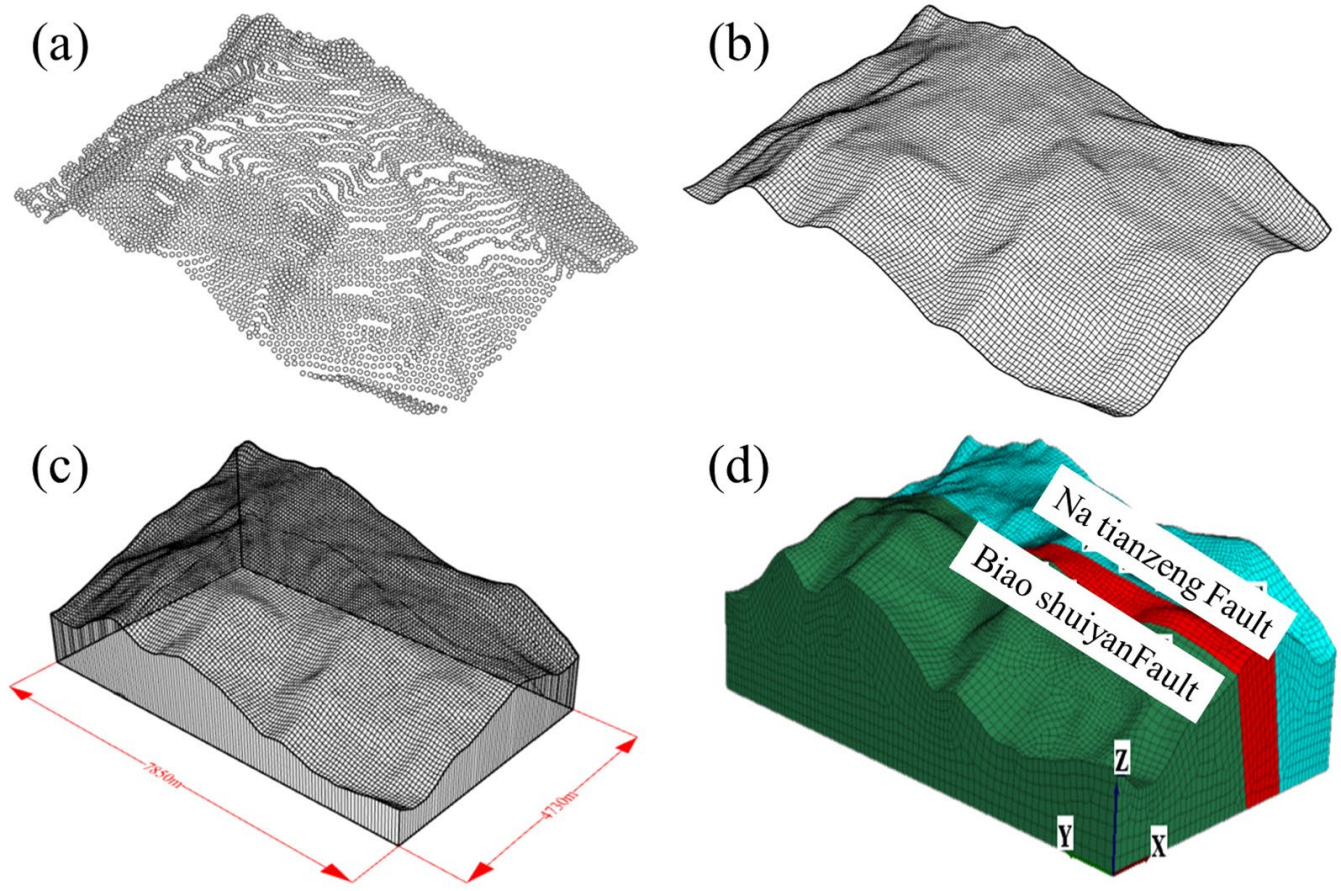
Regional model of geo-stress inversion research; (**a**) contour division; (**b**) MeshPatch; (**c**) entity model; (**d**) FLAC^3D^ model.

Export the contour lines processed in Surfer software to a dxf format file, and use Rhino software to process the dxf file. Divide the contour lines within the study area into points (curve → point object → segment length), with a division size of 50 m, and generate surface point information as shown in Fig. 7a. Based on point information, generate a mesh and apply MeshPatch to the mesh, as shown in Fig. 7b. The line extrusion surface command and the cover command in entity extension generate a solid model, as shown in Fig. 7c. Grid the solid model and export the model information through the Griddle plugin in Rhino. Import the model into FLAC3D and generate the research area model, as shown in Fig. 7d. The model is 7850 m long and 4730 m wide. Assigning different parameters and constitutive relationships to different strata in the model and conducting calculations.

According to tunnel excavation, the rock mass joints are relatively dense. Therefore, to consider the influence of joints on the stability of surrounding rock and the stress of support, this article adopts the Bilinear Strain Softening ubiquitous Joint Model in FLAC^3D^, hereinafter referred to as the Bilinear Model^36,37^. Based on the structural plane information obtained on site (Table 1) and engineering experience analogy, the recommended values of rock and fault physical and mechanical parameter indicators in the tunnel site area are shown in Table 2.

**Table 1:** Physical and mechanical parameters of structural planes.

	Density (kN/m^3^)	Poisson's ratio	Modulus of deformation (GPa)	Cohesion (MPa)	Internal friction angle (°)	Expansion angle (°)
Structural planes	–	–	–	0.02	15	0

**Table 2:** Physical and mechanical parameters of rock and fault.

Rock type	Density (kN/m^3^)	Elastic modulus (GPa)	Poisson’s ratio	Internal friction angle (°)	Cohesion (kPa)
Sandy slate	22.3	1.12	0.28	25	200
Schistosized basalt	23.5	1.01	0.26	23	150
Fault	20.3	0.58	0.30	18	100

### Optimal boundary condition prediction and in-situ stress inversion based on GS-XGBoost algorithm

The in-situ stress values were obtained through the hydraulic fracturing method, and two deep holes (DZ-XCP-02 and DZ-XCP-03) in the middle of the tunnel were subjected to in-situ stress testing. The elevation of borehole DZ-XCP-02 is 2750.01 m, located at DK54 + 310, and the tunnel is buried at a depth of approximately 700 m. The elevation of drilling hole DZ-XCP-03 is 3007.70 m, and the buried depth of the tunnel here is 861.5 m, corresponding to the tunnel stake number DK55 + 606.20. The drilling position is shown in Fig. 12. The results of in-situ stress measurement are shown in Tables 3 and 4.

**Table 3:** DZ-XCP-02 hole hydraulic fracturing in-situ stress measurement results.

Number	Section depth (m)	Fracturing parameters (MPa)					Principal stress value (MPa)			Fracture orientation (°)
		*P*_*b*_	*P*_*r*_	*P*_*s*_	*P*_0_	*σ*_*t*_	*σ*_*H*_	*σ*_*h*_	*σ*_*v*_	
1	327.42–328.22	12.21	10.21	7.71	1.57	2.00	11.36	7.71	8.51	–
2	420.34–421.14	15.62	13.62	9.62	2.48	2.00	12.77	9.62	10.93	N56°W
3	443.59–444.39	12.35	10.35	9.35	2.70	2.00	15.00	9.35	11.53	–
4	462.19–462.99	14.53	13.03	10.53	2.89	1.50	15.68	10.53	12.01	N62°W
5	471.49–472.29	17.62	13.62	10.62	2.98	4.00	15.27	10.62	12.25	–
6	494.74–495.54	17.85	15.35	11.85	3.21	2.50	17.00	11.85	12.86	N54°W

*P*_*b*_ is the fracturing pressure, *P*_*r*_ is the fracturing open pressure, *P*_*s*_ is the instantaneous closing pressure, *P*_*0*_ is the rock pore pressure, *σ*_*t*_ is the rock tensile strength, *σ*_*H*_ is the maximum principal stress, *σ*_*h*_ is the minimum principal stress, and *σ*_*v*_ is the vertical stress.

**Table 4:** DZ-XCP-03 hole hydraulic fracturing in-situ stress measurement results.

Number	Section depth (m)	Fracturing parameters (MPa)					Principal stress value (MPa)			Fracture orientation (°)
		$P_{b}$	$P_{r}$	$P_{s}$	$P_{0}$	$\sigma_{t}$	$\sigma_{H}$	$\sigma_{h}$	$\sigma_{v}$	
1	565.30–566.60	14.54	10.54	10.04	5.15	4.00	14.43	10.04	14.96	N45°W
2	635.20–636.50	16.22	13.72	12.72	5.83	2.50	18.61	12.72	16.81	–
3	690.00–691.30	16.76	15.76	14.26	6.37	1.00	20.65	14.26	18.26	–
4	718.60–719.90	19.54	16.04	14.54	6.65	3.50	20.93	14.54	19.01	–
5	761.20–762.50	22.46	18.46	15.96	7.07	4.00	22.35	15.96	20.14	N26°W
6	836.50–837.80	19.70	17.20	16.20	7.81	2.50	23.59	16.20	22.13	N34°W
7	872.30–873.60	–	20.05	17.55	8.16	–	24.44	17.55	23.08	–
8	887.40–888.70	–	21.20	18.20	8.31	–	25.09	18.20	23.48	N45°W

The depth of borehole DZ-XCP-02 is relatively shallow, and the maximum and minimum horizontal ground stresses buried at depths of 700–1155 m are relatively large, mainly influenced by surface erosion. The erosion process shapes the terrain and topography of the surface, thereby affecting the release and accumulation of local geological stress. The maximum depth of borehole DZ-XCP-03 is 888.70 m, which has good reference value. The average fracture angle of the test results is N 26°–62° W, with an average value of N44° W.

The coordinate system used for calculating the three-dimensional model is generally different from the coordinate system used for calculating the measured in-situ stress. Therefore, coordinate transformation is necessary to determine the maximum horizontal principal stress, minimum horizontal principal stress, and vertical stress directions^38^. Based on existing in-situ stress information, after screening, it has been determined that this article's measured data used for in-situ stress inversion are measurement points 2, 4, and 6 in borehole DZ-XCP-03.

**Figure 8:**
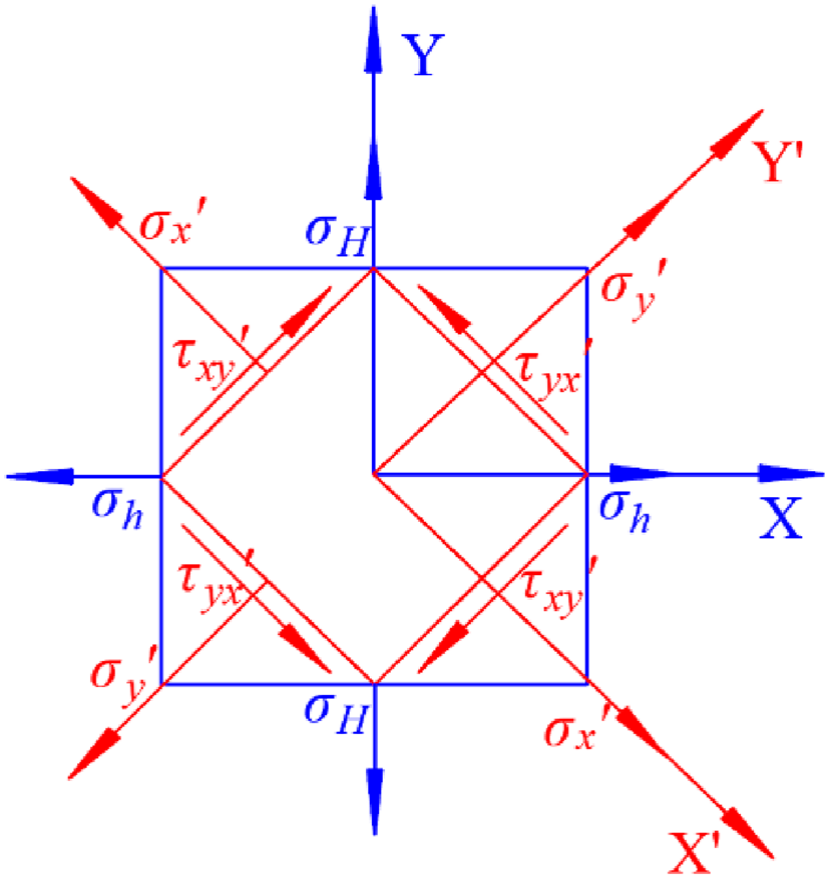
Transformation relationship of principal stress in different coordinate systems.

The coordinate system used for the measured ground stress mentioned in this article is a natural coordinate system, which has an angle with the direction of the tunnel axis. Therefore, it is necessary to consider this angle to convert the measured ground stress into the applicable ground stress value in the model. In Fig. 8, XY is the coordinate system used for measurement, while XʹYʹ is the model coordinate system. The transformation relationship of the principal stress during the transformation from the measurement coordinate system to the model coordinate system is determined by Eq. (19).

19 $$\left\{ {\begin{array}{*{20}l} {\sigma_{x}^{\prime } = \sigma_{x} l_{1}^{2} + \sigma_{y} m_{1}^{2} + 2\tau_{yx} l_{1} m_{1} } \hfill \\ {\sigma_{y}^{\prime } = \sigma_{x} l_{2}^{2} + \sigma_{y} m_{2}^{2} + 2\tau_{yx} l_{2} m_{2} } \hfill \\ {\tau_{xy}^{\prime } = \sigma_{x} l_{1} l_{2} + \sigma_{y} m_{1} m_{2} + 2\tau_{yx} \left( {l_{1} m_{2} + l_{2} m_{1} } \right)} \hfill \\ \end{array} } \right.$$

In the Equation: $\sigma_{x}$, $\sigma_{y}$, $\tau_{yx}$ represents the stress values of each item in the initial coordinate system. $\sigma_{x}^{\prime }$, $\sigma_{y}^{\prime }$, $\tau_{xy}^{\prime }$ is the stress value after being converted to the new coordinate system. $l_{1}$, $l_{2}$, $m_{1}$, $m_{2}$ is the cosine of the direction corresponding to each coordinate axis in the old and new coordinate systems. $\alpha$ is the rotation angle of the old coordinate system in a counterclockwise direction. For the calculation coordinate system of the Haba Snow Mountain Tunnel model, it is known from the characteristics of the regional stress field that $\sigma_{y} { = }\sigma_{H}$, $\sigma_{x} { = }\sigma_{h}$, $\alpha = 44^\circ$. The relationship between $\sigma_{x}^{\prime }$, $\sigma_{y}^{\prime }$, $\tau_{xy}^{\prime }$ in the calculated coordinate system and the measured stress is shown in Eq. 15.

20 $$\left\{ {\begin{array}{*{20}l} {\sigma_{x}^{\prime } = \sigma_{h} \cos^{2} (44^{^\circ } ) + \sigma_{H} \sin^{2} (44^{^\circ } )} \hfill \\ {\sigma_{y}^{\prime } = \sigma_{h} \sin^{2} (44^{^\circ } ) + \sigma_{H} \cos^{2} (44^{^\circ } )} \hfill \\ {\tau_{xy}^{\prime } = \left( {\sigma_{h} + \sigma_{H} } \right)\sin (44^{^\circ } )\cos (44^{^\circ } )} \hfill \\ \end{array} } \right.$$

The above borehole geo-stress information is converted through coordinate transformation into the tunnel model coordinate system. Figure 8 is a schematic diagram of stress direction transformation, and the transformed geo-stress information is shown in Table 5.

**Table 5:** Selected geostress information after coordinate transformation.

Number	Drill hole number	Buried depth (m)	$\sigma_{XX}$(MPa)	$\sigma_{YY}$ (MPa)	$\sigma_{ZZ}$ (MPa)	$\tau_{XY}$ (MPa)
1	DZ-XCP-03	635.75	− 15.56	− 15.77	− 16.81	− 15.66
2	DZ-XCP-03	690.65	− 17.46	− 17.46	− 18.26	− 17.46
3	DZ-XCP-03	719.25	− 17.85	− 17.62	− 19.01	− 17.72
4	DZ-XCP-03	761.85	− 19.38	− 18.93	− 20.14	− 19.11
5	DZ-XCP-03	837.15	− 20.28	− 19.51	− 22.13	− 19.79

From Table 5, it can be seen that for the coordinate system of the geo-stress inversion model, the range of horizontal lateral pressure coefficients is $\lambda_{X}$ = 0.76–0.96, $\lambda_{Y}$ = 0.88–1.15, and the range of shear stress in the XY direction is $\tau_{XY}$ = − 15.66 to − 19.79 MPa. Based on the measured values of the weight of the surrounding rock under in-situ stress and the actual weight of the surrounding rock, it can be concluded that the weight compensation value $\lambda_{G}$ = 1.11–1.33. Therefore, different boundary conditions are designed as shown in Table 6, and the optimal boundary conditions are obtained through the GS-XGBoost algorithm as shown in Table 7, with $\lambda_{G}$ = 1.23, $\lambda_{X}$ = 0.94, $\lambda_{Y}$ = 1.12, $\tau_{XY}$ = 18.21 MPa.

**Table 6:** Displacement boundary condition test group U30 (30^4^).

Number	$\lambda_{G}$	$\lambda_{X}$	$\lambda_{Y}$	$\tau_{XY}$ (MPa)
1	1.110	0.760	0.880	15.660
2	1.118	0.767	0.889	15.802
…		…	…	…
29	1.322	0.953	1.141	19.648
30	1.330	0.960	1.150	19.790

**Table 7:** Optimal stress boundary condition.

Boundary condition	$\lambda_{G}$	$\lambda_{X}$	$\lambda_{Y}$	$\tau_{XY}$ (MPa)
Predictive value	1.23	0.94	1.12	18.21

The results of in-situ stress inversion are shown in Table 8. Table 8 shows that the difference between the inversion results of ground stress and the measured results is between − 15 and 19%, which can, to some extent, restore the distribution of the ground stress field in the tunnel area. The difference may be because the lithology and geological structure of the tunnel area cannot be obtained through a small amount of drilling, and the distribution of the lithology and geological structure is relatively complex. Only the main strata and structures were considered in the simulation.

**Table 8:** Summary of geo-stress inversion results.

Drill hole number	Measurement point number	Buried depth (m)	Stress component	Self weight (Pa)	X-direction compressive stress (Pa)	Y-direction compressive stress (Pa)	XY shear stress (Pa)	Measured value (Pa)	Inversion value (Pa)	Measured to calculated difference ratio (%)
DZ-XCP-03	2	635.75	$\sigma_{X}$	− 6.55 × 10^6^	− 8.36 × 10^4^	− 1.57 × 10^4^	− 5.68 × 10^3^	− 1.58 × 10^7^	− 1.38E + 07	19
			$\sigma_{Y}$	− 7.85 × 10^6^	− 2.53 × 10^4^	− 6.42 × 10^4^	− 6.44 × 10^3^	− 1.56 × 10^7^	− 1.69E + 07	8
			$\sigma_{Z}$	− 1.48 × 10^6^	− 7.44 × 10^0^	− 2.21 × 10^3^	− 4.43 × 10^3^	− 1.68 × 10^7^	− 1.87E + 07	6
			$\tau_{XY}$	− 4.08 × 10^6^	1.71 × 10^3^	− 1.58 × 10^3^	− 1.09 × 10^5^	− 1.57 × 10^7^	− 1.24 × 10^7^	11
DZ-XCP-03	4	719.25	$\sigma_{X}$	− 7.07 × 10^6^	− 8.59 × 10^4^	− 1.63 × 10^4^	− 4.89 × 10^3^	− 1.78 × 10^7^	− 1.90 × 10^7^	9
			$\sigma_{Y}$	− 8.30 × 10^6^	− 2.64 × 10^4^	− 6.48 × 10^4^	− 5.85 × 10^3^	− 1.76 × 10^7^	− 1.63 × 10^7^	− 1
			$\sigma_{Z}$	− 1.66 × 10^7^	9.41 × 10^2^	− 1.72 × 10^3^	− 4.57 × 10^3^	− 1.90 × 10^7^	− 1.58 × 10^7^	4
			$\tau_{XY}$	− 4.31 × 10^5^	1.76 × 10^3^	− 1.51 × 10^3^	− 1.11 × 10^5^	− 1.77 × 10^7^	− 1.55 × 10^7^	0
DZ-XCP-03	6	837.15	$\sigma_{X}$	− 7.19 × 10^6^	− 8.63 × 10^4^	− 1.65 × 10^4^	− 5.23 × 10^3^	− 2.00 × 10^7^	− 2.31 × 10^7^	− 1
			$\sigma_{Y}$	− 8.45 × 10^6^	− 2.65 × 10^4^	− 6.51 × 10^4^	− 6.02 × 10^3^	− 1.98 × 10^7^	− 1.72 × 10^7^	− 11
			$\sigma_{Z}$	− − 1.71 × 10^7^	1.04 × 10^3^	− 1.62 × 10^3^	− 4.60 × 10^3^	− 2.21 × 10^7^	− 2.13 × 10^7^	− 9
			$\tau_{XY}$	− 4.34 × 10^5^	1.59 × 10^3^	− 1.44 × 10^3^	− 1.11 × 10^5^	− 1.99 × 10^7^	− 1.84 × 10^7^	− 10

### Analysis of inversion results of ground stress field

**Figure 9:**
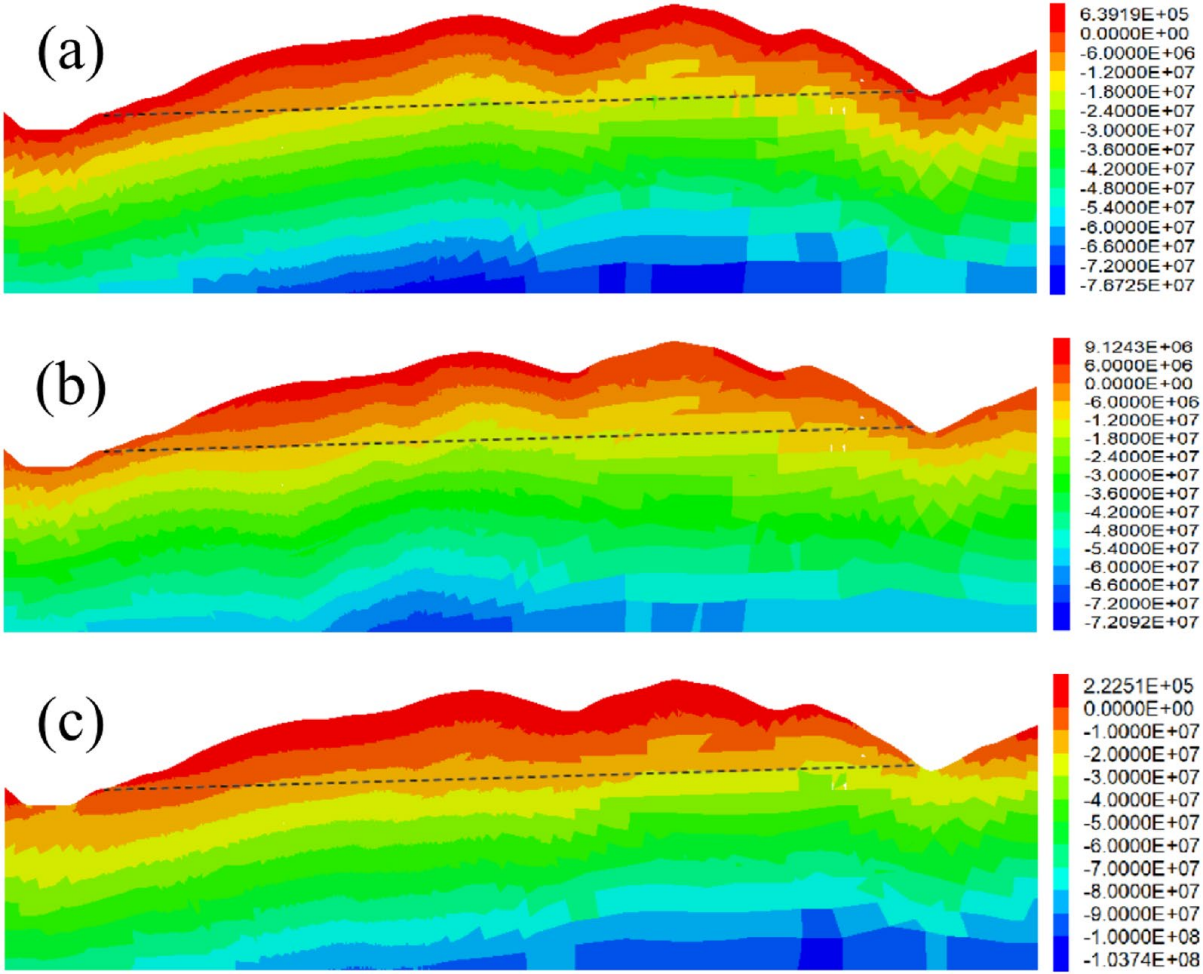
Geo-stress field in tunnel area: (**a**) Vertical stress; (**b**) Minimum horizontal principal stress; (**c**) Maximum horizontal principal stress.

**Figure 10:**
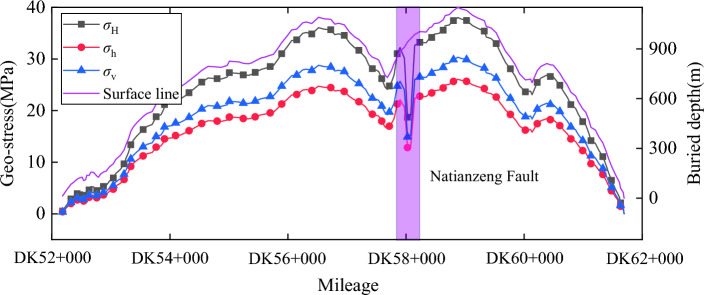
Ground stress distribution along the tunnel axis.

The geo-stress information on the tunnel axis is extracted by inverting the geo-stress field in the tunnel area. After coordinate transformation, it is shown in Figs. 9 and 10.

From Figs. 9 and 10, it can be seen that the tunnel ground stress increases with the increase of burial depth, with the maximum horizontal ground stress of 38.03 MPa and the maximum, minimum horizontal ground stress of 26.07 MPa, located near the tunnel section DK58 + 877. In addition, according to the in-situ stress information, the pattern of the in-situ stress field near the fault is that the in-situ stress on the upper wall of the fault increases, and the in-situ stress on the lower wall decreases.

## Determination of large deformation level of Haba Snow Mountain Tunnel

**Figure 11:**
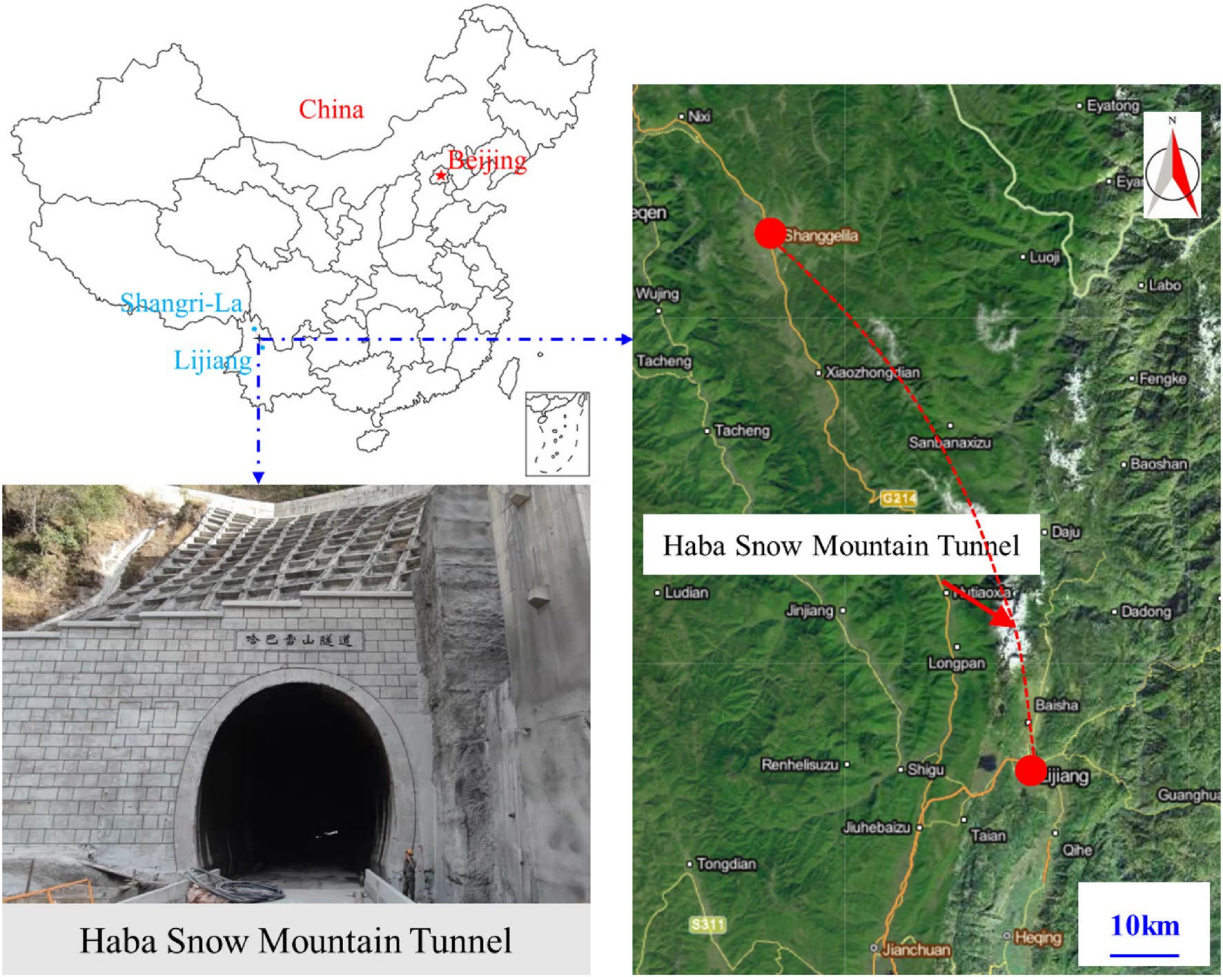
Location of Lijiang–Shangri-La railway and Haba Snow Mountain Tunnel.

**Figure 12:**
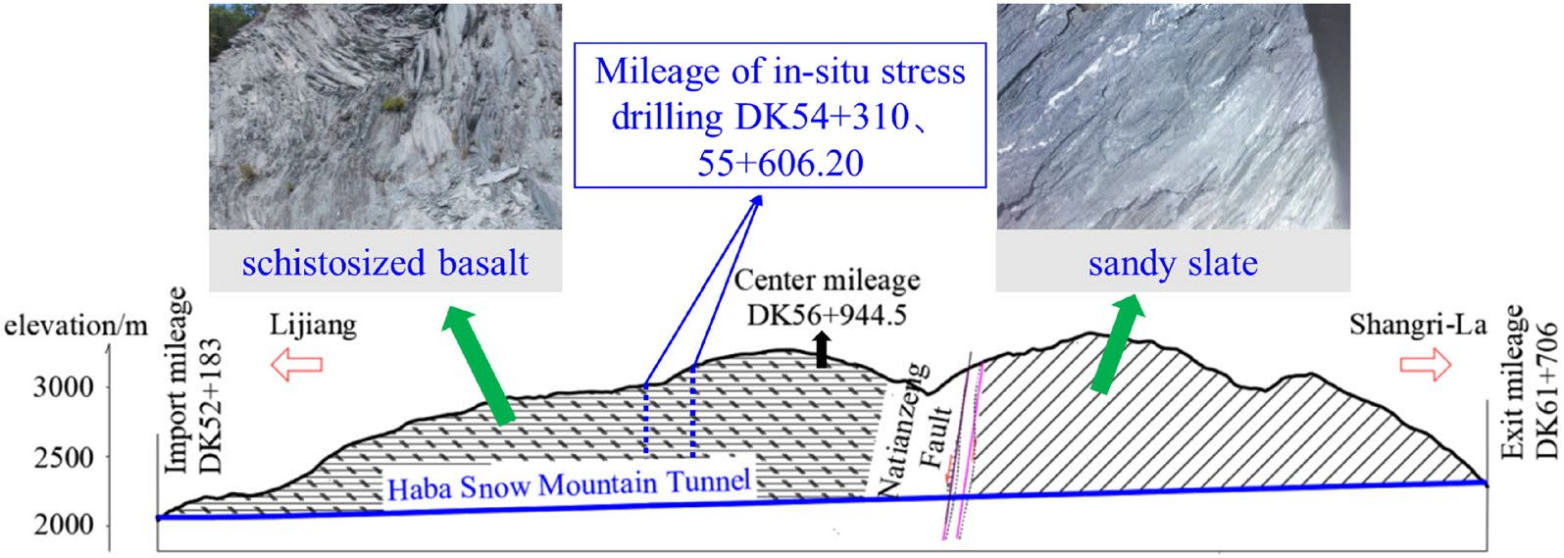
Geological profile of Haba Snow Mountain Tunnel.

Haba Snow Mountain is located on the southeast edge of the Qinghai Tibet Plateau, in the middle section of the Hengduan Mountains, on the left bank of the Jinsha River, and belongs to the plateau tectonic erosion landform area^39^. The mountain runs in a nearly north–south direction, with a terrain low on the left and high on the right, and is located in the eroded platform of the Chongjiang River and Jinsha River. The ground elevation ranges from 2050 to 3400 m, with significant terrain fluctuations and a natural cross slope of 15°–40°, with local cliffs. The Haba Snow Mountain Tunnel is a mountain crossing tunnel with well-developed vegetation on the slope, mostly pine forests. The tunnel site area only has soil access roads that can reach the entrance and exit of the tunnel, and most of the transverse tunnel exits are transported on average. The total length of the tunnel is 9523 m, with the maximum burial depth (1155 m) at the tunnel mileage of approximately DK58 + 900. The surrounding rock of the tunnel is mainly composed of schistose basalt and sandy slate. The basic engineering information of the Haba Snow Mountain Tunnel is shown in Fig. 11, and the geological profile is shown in Fig. 12.

**Figure 13:**
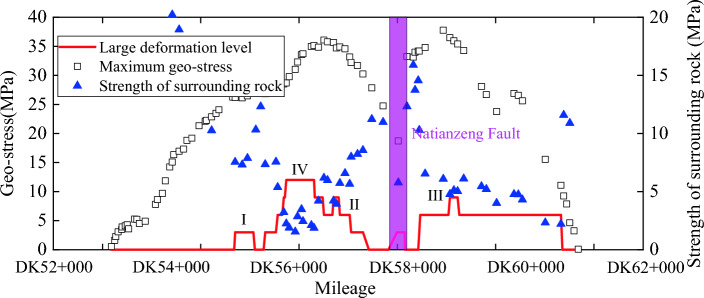
Grade determination of large deformation of Haba Snow Mountain Tunnel.

The classification of large deformation of Haba Snow Mountain Tunnel adopts the classification standard for the deformation grade of compressible surrounding rock tunnels. This classification method mainly considers the strength and stress ratio of the rock mass (*G*_N_), as shown in Table 9. According to the on-site testing results of severe large deformation sections, the rock mass strength of the Haba Snow Mountain Tunnel is 1.3–3.7 MPa. According to the inversion results of ground stress, the tunnel ground stress is about 26.07–38.03 MPa. Therefore, the rock mass strength stress ratio of the severe large deformation section of the tunnel is 0.03–0.09, belonging to level IV soft rock large deformation. Other paragraphs have significant deformations of levels I, II, and III. Therefore, the determination of the large deformation level of the Haba Snow Mountain Tunnel is shown in Fig. 13.

**Table 9:** Classification standard for large deformation of squeezing tunnel.

Large deformation level	I	II	III	IV
$G_{N} = R_{cm} /\sigma_{\max }$	0.30 ≥ G_N_ > 0.20	0.20 ≥ G_N_ > 0.15	0.15 ≥ G_N_ > 0.10	G_N_ ≤ 0.10

Figure 13 shows that the Haba Snow Mountain Tunnel has large deformation problems of Levels I, II, III, and IV. The maximum in-situ stress in the Level I large deformation section is between 18.71 and 34.17 MPa, and the strength of the surrounding rock is between 5.78 and 10.29 MPa. The maximum in-situ stress in the Level II large deformation section is between 11.08 and 37.76 MPa, and the strength of the surrounding rock is between 2.21 and 6.54 MPa. The maximum in-situ stress in the Level III large deformation section is between 28.52 and 36.47 MPa, and the strength of the surrounding rock is between 3.23 and 5.11 MPa. The maximum in-situ stress in the Level IV large deformation section is between 28.70 and 35.14 MPa, and the strength of the surrounding rock is between 1.55 and 3.48 MPa, mainly concentrated near DK56 + 000. Level III and IV large deformations are generally accompanied by higher ground stress (above 28 MPa) and smaller surrounding rock strength (below 6.0 MPa). The distribution of surrounding rock strength in the direction of the tunnel axis shows a clear "W" shape, opposite to the surface elevation "M" shape. Based on the Haba Snow Mountain Tunnel's terrain on the banks of the Jinshan River, it is inferred that the mountain may be affected by geological structures on both sides, causing more severe compression of the tunnel surrounding rock at the mountain peak.

## Conclusion

Based on the GS-XGBoost Algorithm, the inversion of the ground stress in the Haba Snow Mountain Tunnel shows that the difference between the inversion results and the measured results is − 15 to 19%, which can to some extent restore the distribution of the ground stress field in the tunnel area. The tunnel ground stress increases with burial depth, with a maximum horizontal ground stress of 38.03 MPa and a minimum horizontal ground stress of 26.07 MPa, located near the tunnel section DK58 + 877. The Haba Snow Mountain Tunnel has large deformation problems of Level I, II, III, and IV. Level III and IV large deformations are generally accompanied by higher ground stress (above 28 MPa) and smaller surrounding rock strength (below 6.0 MPa). The distribution of surrounding rock strength in the direction of the tunnel axis shows a clear "W" shape, opposite to the surface elevation "M" shape. Based on the Haba Snow Mountain Tunnel's terrain on the banks of the Jinshan River, it is inferred that the mountain may be affected by geological structures on both sides, causing more severe compression of the tunnel surrounding rock at the mountain peak.

The trend of combining intelligent algorithms with in-situ stress inversion methods is evolving. However, further research is needed on the accuracy of the inversion of in-situ stress field range and the density of in-situ stress measured boreholes. In addition, the impact of the accuracy of in-situ stress testing methods on the results of in-situ stress inversion is also worth further exploration.

## Data Availability

The datasets used and analyzed during the current study are available from the corresponding author upon reasonable request.
